# Bioactivity of Biphasic Calcium Phosphate Granules, the Control of a Needle-Like Apatite Layer Formation for Further Medical Device Developments

**DOI:** 10.3389/fbioe.2019.00462

**Published:** 2020-01-28

**Authors:** Cyril d’Arros, Thierry Rouillon, Joelle Veziers, Olivier Malard, Pascal Borget, Guy Daculsi

**Affiliations:** ^1^INSERM, UMR 1229, Regenerative Medicine and Skeleton, ONIRIS, Université de Nantes, Nantes, France; ^2^Biomatlante – Advanced Medical Solutions Group plc, Vigneux-de-Bretagne, France; ^3^UFR Odontologie, Université de Nantes, Nantes, France; ^4^PHU4 OTONN, CHU de Nantes, Nantes, France; ^5^INSERM, UMS 016, CNRS 3556, Structure Fédérative de Recherche François Bonamy, SC3M Facility, CHU de Nantes, Université de Nantes, Nantes, France; ^6^Service d’Oto-Rhino-Laryngologie et de Chirurgie Cervico-Faciale, PHU4 OTONN, CHU de Nantes, Nantes, France

**Keywords:** biphasic calcium phosphate, bioactive ceramic, apatitic needle-like layer, bioactivity, apatitic TCP

## Abstract

Biphasic calcium phosphate (BCP) bioceramics (hydroxyapatite/tricalcium phosphate, or HA/TCP) for tissue engineering and drug delivery systems is a unique know-how. A mechanical mixture of HA and TCP does not lead to such bioactive ceramics. The wet elaboration conditions of calcium-deficient apatite (CDA) or CDHA, followed by sintering, converts it into TCP and HA. The dissolution precipitation of nano-sized needle-like crystals at the surface of BCP occurs on time at body temperature. Combining several technics of characterization [scanning electron microscopy (SEM), transmission electron microscopy (TEM), energy-dispersive x-ray spectroscopy (EDX), Brunauer-Emmett-Teller method (BET), chemical analysis, x-ray diffraction (XRD), Fourier transformed infrared spectroscopy (FTIR)], we demonstrated an evolution on time of the HA/β-TCP. The current paper describes the crystallographic evolution of initial β-TCP rhombohedral crystallographic structure to microsized needle-like layer corresponding to apatitic TCP form. This phenomenon leads to an increase of the HA/TCP ratio, since hexagonal apatitic TCP is similar to hexagonal HA. However, the Ca/P ratio (reflecting the chemical composition HA/TCP) remains unchanged. Thus, the high reactivity of BCP involves dynamic evolution from rhombohedral to hexagonal structure, but not a chemical change. The dynamic process is reversible by calcination. These events are absolutely necessary for smart scaffolds in bone regeneration and orthobiology.

## Introduction

Many clinical situations require materials to restore and regenerate the bone. In spite of large innovations during the last 30 years (Wagner et al., 2018), the optimization of synthetic bone substitutes is still required to have an efficient clinical alternative to the gold standard, the autograft, which has several limitations frequently described in the literature (Heary et al., 2002; Silber et al., 2003). An important part of synthetic bone graft development in the last few decades concerns calcium phosphate (CaP) bioceramics as a replacement for auto- and allografts (Giannoudis et al., 2005; Dorozhkin, 2010; Habraken et al., 2016), or xenografts (Daculsi et al., 2006). This can be achieved by the *in vivo* formation of a carbonated apatite layer allowing for a strong interaction of the implanted CaP materials with the host bone as demonstrated 30 years ago (Daculsi et al., 1990). The recent evaluation (Bohner and Miron, 2019) of the apatitic precipitation clearly confirms that this process is a prerequisite for a bioactive CaP bioactive materials. To predict the bonding of synthetic materials to a living bone, an *in vitro* method was assessed based on the observation of apatite layer onto the surface of synthetic materials immerged in a simulated body fluid, with ion concentrations nearly equal to those of human blood plasma (Kokubo and Takadama, 2006). This property was further defined as bioactivity.

Biphasic calcium phosphates (BCP) are largely used in clinical situations. The ratio of hydroxyapatite (HA) and β-tricalcium phosphate (β-TCP) is a crucial parameter concerning the bioactivity of this kind of bone substitute, due to different solubilities between both compounds of BCP (Lin et al., 2003; Dorozhkin, 2010). Lower sintering temperature (<1200°C) respects the chemical composition and the high reactivity of the bioceramics in terms of dissolution/precipitation required for effective bone regeneration technologies (Gauthier et al., 1999; Habibovic et al., 2006a). In addition, the macro- and microporosity of CaP bioceramics play a fundamental and key role in the osteoinduction process (Daculsi and Layrolle, 2004; LeGeros, 2008; Champion, 2013; Eliaz and Metoki, 2017).

MBCP^®^+ BCP synthetic bone graft is currently used in clinical trials as a scaffold for tissue engineering strategy. Indeed *in vitro* and *in vivo* experiments testing autologous mesenchymal stem cells associated with these BCP granules displayed excellent results in bone regeneration (Cordonnier et al., 2010; Brennan et al., 2014). A clinical trial’s results demonstrated a promising future for the use of autologous stem cells combined with highly bioactive BCP granules (Gjerde et al., 2018; Gómez-Barrena et al., 2019).

New scaffolds designs have to be more suitable for a tissue engineering approach, improving usability for surgeons at the same time. It appears that ease of use and handling of injectable or moldable paste bone medical devices are much anticipated (Bohner, 2010). One of the most important goals for new synthetic bone substitute development is to facilitate its handling for the surgeons while at the same time maintaining the bioactivity of highly reactive CaP bioceramics. Using hydrophilic carriers with CaP granules for pastes and putties can lead to interaction with mineral phase over time (Bourges et al., 2001; Schmitt et al., 2002; Davison et al., 2012).

It is therefore of prime importance to understand how it is possible to get a bioactive layer on BCP materials and what the physicochemical composition of this layer is to better control its formation, in order to develop the next generation of synthetic bone graft materials.

## Materials and Methods

### BCP Samples

#### Washing, Drying, and Sintering

The MBCP^®^+ bioceramic (Biomatlante SA, France) was used in this study. This commercial BCP is an intimate mixture at the molecular level of 20% of HA and 80% of β-TCP sterilized by beta irradiation (25 kGy).

Three batches of MBCP^®^+ granules were considered and labeled BCP1, BCP2, and BCP3, corresponding to batch numbers 0716J116, 0916J216, and 1016J216, respectively. Five hundred micrograms of each batch was washed separately three times for 5 min ultrasonically in distilled water, then dried in an oven at 200°C for 4 h and labeled _W&D (for washed and dried). Ten grams of each batch was then sintered again at low temperature following Biomatlante SA’s validated process (<1100°C) and _W&D_S (for washed, dried, and sintered). Denominations of all the samples used in the study are summarized in Supplementary Data S1.

Granules (1–2 mm) were analyzed using scanning electron microscopy (SEM), x-ray diffraction (XRD), atomic absorption spectrometry (AAS) for the Ca/P ratio, and Fourier transformed infrared spectroscopy (FTIR) analyses. The Brunauer-Emmett-Teller (BET) method for specific surface area (SSA) analyses was performed on 0.5 to 1 mm granules of BCP2.

#### Effect of Higher Sintering Temperature

To investigate the effect of higher sintering temperature on the bioactivity, two samples (1 g of 0.5 to 1 mm granules each) were examined: one control (BCP_0) and another one (BCP_1200) whose microstructure was modified by an additional sintering step at higher temperature (1200°C/5 h).

Each batch was washed and dried as previously described (labeled _W&D) with more efficiency due to the small amount considered (1 g instead of 500 g). Both samples were then stored in wet conditions at 37°C for 2 months and freeze-dried to remove residual water instead of conventional drying in the oven (labeled _W&37/2M). Denominations of all the samples used in the study are summarized in Supplementary Data S1.

### Characterization

#### Scanning Electron Microscopy

Biphasic calcium phosphate granules were coated with gold using a Desk V Sputter (Denton Vacuum), and the surface topographies were examined by secondary electrons using a SEM (LEO 1450VP) at an acceleration voltage of 10 keV and 30 mA.

#### Specific Surface Area

The SSA was determined by the BET method. The BET experiment was measured by nitrogen gas adsorption on a Micromeritics 3-FLEX equipment. Samples were weighed (about 100 mg) and degassed in vacuum conditions (10^–3^ mbar) at 150°C for 24 h. Following this step, the samples were precisely weighed and their SSAs were calculated from the range of relative pressure of adsorption–desorption isotherms by 0.05–0.2. The unit of BET SSA is m^2^/g.

#### X-Ray Diffraction

The identification phase of BCP materials was determined by analyzing the x-ray powder diffraction (XRD) data recorded using a Philips PW 1830 generator equipped with a copper x-ray tube, a vertical PW 1050 (θ/2θ) goniometer, and a PW 1711 Xe detector. Measurements were performed using the CuKα radiation (Ni filtered) and recorded in a step-by-step mode of 2θ from 3° to 70° with a step 2θ of 0.03° and a counting time per step of 3 s. The HA/β-TCP ratio (or hexagonal/rhombohedral phases ratio) of the BCP powder (without and with an apatitic surface layer formation, respectively) was determined from XRD measurements based on the ratio of peak height of the most intense reflection of each phase, corresponding to the (0 2 10) reflection at 2θ≈ 31.0° for the rhombohedral phase (β-TCP, ICDD-PDF database ref. 009-0169) and to the (2 1 1) reflection at 2θ≈ 31.7° for the hexagonal phase (HA, ICDD-PDF database ref. 009-0432).

#### CaP Analyses

The dosages of calcium and phosphates allowed for determining the Ca/P ratio are particularly important concerning the characterization of a BCP.

Calcium analysis was performed by AAS using a Thermo Scientific ICE 3300 spectrometer. Around 250 mg of BCP powder was dissolved in around 15 ml of concentrated nitric acid–HNO_3_ (65%) and then diluted with deionized water and completed with lanthanum chloride in order to obtain a final solution of around 2 mg/L of calcium with 1% HNO_3_ and 1% lanthanum (in order to avoid interference phenomena in the flame). Measurements of absorbance were performed at 422.7 nm using a calcium lamp and an air/acetylene flame. Calibration was performed from solutions of different concentrations of calcium with 1% HNO_3_ and 1% lanthanum, and prepared from a 1 g/L of certified calcium solution (ref. 86667.260 VWR).

The phosphate ions (PO_4_^3–^) concentration was determined by colorimetry using a PerkinElmer Lambda25 UV/Vis spectrometer. Vanadate–molybdate reagent (ref. 1.08498.0500; Merck) was used to determine the phosphate concentration. Around 100 mg of BCP powder was dissolved in 5 ml of concentrated nitric acid–HNO_3_ (65%) and then diluted with deionized water and adjusted with Titripur^®^ Sodium Hydroxide solution 1 N (ref. 1.09137.1000; Merck) or Titripur^®^ Sulfuric Acid 1 N (ref. 1.09072.1000; Merck) in order to obtain a neutral solution (pH around 7) with an amount of phosphates corresponding to approximatively 100 mg/L of BCP sample. Measurements of absorbance were performed at 405 nm, 15 min after adding and agitating 5 ml of the sample solution with 1 ml of the V-M reagent. Calibration was performed from solutions of different concentrations of phosphate, prepared by dilutions from 1 g/L of phosphate solution (potassium dihydrogen phosphate anhydrous, ref. 1.05108.050; Merck).

#### Fourier Transformed Infrared Spectroscopy

Pellets made of around 1–2 mg of crushed samples, mixed with 300 mg of KBr and pressed, were prepared, and infrared absorption spectra by transmission were collected in the 400- to 4000-cm^–1^ spectral range with a resolution of 4 cm^–1^ and an accumulation of 64 scans using a Nicolet Magnat II 550 FTIR spectrometer.

#### Transmission Electron Microscopy

A small amount of BCP sample was shaken vigorously in less than 1 ml of pure-grade ethanol, after which the larger particles descended to the bottom. A few drops of the supernatant with needle suspension were deposited on a nickel grid for transmission electron microscopy (TEM) and covered with holey carbon film. After solvent evaporation, the grid was observed by TEM using a 1010 JEOL electron microscope operating at an accelerating voltage of 100 kV. Selected area electron diffraction (SAED) patterns were performed at 100 kV, with a double-tilt holder, and the diffraction constant was calibrated using an evaporated aluminum film as a standard. Energy-dispersive x-ray spectroscopy (EDX) measurements were performed at 100 kV using an Oxford Instruments Link ISIS spectrometer, equipped with an ATW2 ultrathin window (energy resolution: 142 eV at 5.9 keV).

The mean value of the Ca/P atomic ratio of needles was determined from EDX microanalyses performed on more than 10 different needles. Quantitative analyses were obtained on the basis of thin film approximation, and the calcium and phosphorus experimental Cliff–Lorimer K factors were standardized from analyses performed under the same conditions on β-TCP [β-Ca_3_(PO_4_)_2_] and octa calcium phosphate [Ca_8_H_2_(PO_4_)_6_⋅5H_2_O] standards. Schematic 2D representations of the reciprocal lattice compared to ED patterns were simulated using CaRIne Crystallography 3.1 software with a hexagonal crystallographic cell corresponding to the HA crystalline structure (a = 9.42 Å, c = 6.88 Å, space group P 6_3_/m).

## Results and Discussion

**FIGURE 1:**
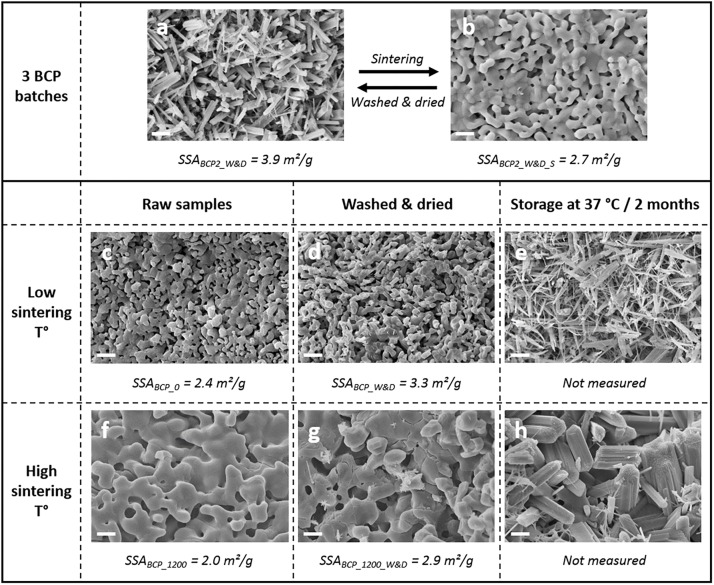
Scanning electron microscopy (SEM) observations (magnification ×5000, scale bar of 2 μm) on the effect of the washing and drying on biphasic calcium phosphate (BCP) granules **(a)** and BCP surface after a new low-temperature sintering step **(b)**. Comparison of the original BCP granules in different conditions (**c**, sample BCP_0) and the same granules after a new sintering step at 1200°C/5 h (**f**, sample BCP_1200), with the evolution of surface after washing and drying for a small amount (**d**,**g**, respectively; BCP_W&D and BCP_1200_W&D for original and 1200°C sintered BCP), and after washing and storage at 37°C for 2 months before freeze drying (**e**,**h**, respectively; for BCP_W&37/2M and BCP_1200_W&37/2M)., using the Brunauer-Emmett-Teller (BET) method to determine the specific surface area values of washed and dried granules and sintered at low and high sintering temperatures of MBCP^®^+ granules **(a**–**d**,**f**,**g)**.

Scanning electron microscopy images illustrate the easy capacity of BCP granules to form an apatitic layer on the surface. Indeed a needle-like structure recovers the whole surface of BCP just after being washed in distilled water and dried in an oven. This kind of result is often sought-after and obtained in more critical situations by using an autoclave (De Groot-Barrere et al., 2015; Duan et al., 2019) in order to improve the bioactivity of the BCP biomaterial. Duan et al. (2019) described higher protein adsorptions and an improved *in vivo* bone regeneration with the presence of needles on the BCP bone graft compared to standard microporous samples. This study shows that this phenomenon is a reversible one since the initial surface is recovered after a new sintering step (Figures 1a,b) as described in a previous study by Bohne et al. (1993). The reproducibility is confirmed on three batches of the BCP (representative images in Figures 1a,b). A higher sintering temperature leads to the increase of grain size of the bioceramic (Figures 1c,f) as extensively described in the literature (Habibovic et al., 2006b). Washing a small amount of BCP granules in distilled water and drying at 200°C (BCP_W&D, Figure 1c) lead to a less important surface modification in comparison to the first condition (BCP1_W&D, BCP2_W&D, and BCP3_W&D illustrated in Figure 1a). This is also observed on the surface of granules sintered at a higher temperature (BCP_1200_W&D, Figure 1g). The most interesting part of this study concerns the formation of a needle-like surface of BCP granules not only at high temperature during drying but also at 37°C near physiological conditions (Figures 1e,h). The size of the newly formed needle-like crystals is proportional to the initial grain size of the BCP (at low and higher sintering temperatures). This high chemical reactivity could explain the important bioactivity of BCP granules and classify it as a smart scaffold (Daculsi, 2015; Zhang et al., 2018). Numerous publications and reviews in the field of bioceramics indicated that the microporosity for bioactive resorbable material is a crucial parameter (Lin et al., 2003; Habibovic et al., 2006a; LeGeros, 2008). This study points out the importance of other parameters to keep a highly reactive BCP (Figure 1h still shows a modification of surface layer on BCP with a high sintering temperature, which decreases the quantity and size of the microporosity). The synthesis method by wet precipitation of BCP leads to an intimate mixing of HA and β-TCP phases at a submicronic level (Miramond et al., 2014b), and then improves this high reactivity exposed in the present study. The role of lattice defects in the case of low sintering temperature should also explain the high reactivity of the BCP as demonstrated in 2005 (Daculsi et al., 2005).

The washing and drying steps increase the SSA of about 30% for the three kinds of materials tested (BCP2 of the 500 g washed and dried batch, BCP of the 1 g washed and dried batch, and BCP sintered at high temperature) with about 4 m^2^/g for 0.5–1 mm MBCP^®^+ granules. The high sintering temperature of 1200°C leads to a decrease of the SSA compared to that of the original BCP granules. The values of this analysis correspond to the ones found in the literature with about 2.5 m^2^/g (Miramond et al., 2014a; Duan et al., 2018). A coherent correlation is possible between SEM observations (Figures 1a–d,f,g) and SSAs. The increase of the SSA values should be more important with smaller granule sizes because of the more important proportion of the surface compared to the mass of the material. In this way, it could be possible to have a control on the BET SSA with the size of the granules and the conditions of washing and drying. A high SSA of granules is a key parameter for the design of drug delivery systems for peptide adsorption and long time delivery as described for spine fusion (Smucker et al., 2008).

**FIGURE 2:**
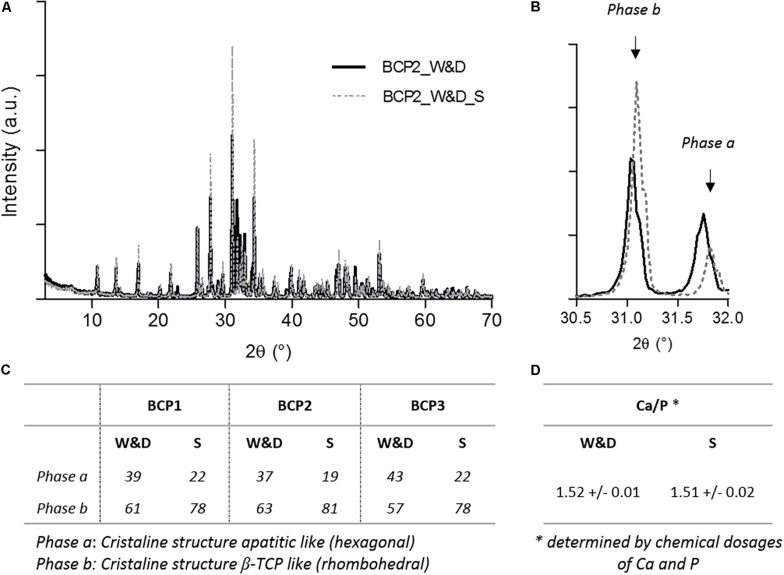
X-ray diffraction (XRD) analyses and calcium phosphate dosages of BCP granules in both conditions. **(A)** Representative graph of XRD analysis after washing and drying, BCP2_W&D, and after a new sintering step, BCP2_W&D_S). **(B)** Focused graph on both peaks for quantification of phases a and b. Table summarizing **(C)** the results of the quantification of both phases a and b for three batches, and **(D)** the results of the Ca/P ratio determined by chemical dosages of three batches (BCP1, BCP2, and BCP3).

The XRD analysis demonstrated different patterns between the washed and dried granules and the granules just after new sintering step. A rapid analysis would lead to a wrong interpretation and a ratio HA/β-TCP of about 40/60 for the conditions after washing and drying. However, the analysis after a sintering step (1000°C/15 h as required by ISO 13779-3 standard) (Afnor, 2019) shows the expected 20/80 (HA/β-TCP) ratio announced by the manufacturer. These repeated results of the three batches are summarized in Figure 2. The calcium and phosphate dosages confirm that the Ca/P ratio is not affected by the washing and drying process for the three batches used in the first part of the study. The increase of the hexagonal phase coupled with the SEM observations can be related to the apatitic surface formation on the surfaces of the granules as described in the literature (Mortier et al., 1989a, b). The observed needle-like structure seems to be exactly the same as that found on the BCP granules of our study. The evident explanation of this observed phenomenon should be the dissolution and precipitation of the BCP leading to a deposited CDA in the needle-like TCP apatitic layer due to a higher solubility of β-TCP compared to HA as extensively described in the literature (Dorozhkin, 2010). This specific chemical composition of MBCP^®^ technology granules could explain the high bioactivity and the excellent osteointegration of this biomaterial after *in vivo* implantation (Daculsi et al., 1990; Rohanizadeh et al., 1999). Moreover, this dynamic evolution on the surface of the BCP granules without any additional ions, contrary to the method proposed by Kokubo and Takadama (2006) for the assessment of the bioactivity of biomaterials with SBF solution, demonstrates the smart response of this BCP at physiological temperature. The immersion in SBF solution also induces the precipitation of an apatitic layer on the MBCP^®^ granules and a high protein adsorption capacity (Duan et al., 2018). In the ratio 20/80 (HA/β-TCP) of MBCP^®^+ granules, the high proportion of the most soluble compound (β-TCP) in this BCP should improve the important bioactivity of this bioceramic. The presence of HA in BCP seems to be very important and could play a role of a catalyzer for the crystalline growth of apatitic layer with hexagonal phase structure (Rohanizadeh et al., 1999; De Groot-Barrere et al., 2015). This process of dissolution and precipitation has been described in high-resolution TEM (Hr TEM) as a secondary nucleation process and heteroepitaxic crystal growth (Daculsi et al., 1990).

The FTIR analysis comparing conditions after a simple washing and drying (BCP_W&D) and the same BCP after an additional sintering step (BCP_W&D_S) shows that the needle-like surface should probably be an apatitic TCP layer. As displayed by equation 1, the reaction of β-TCP with water gives apatitic TCP with new easily identifiable groups on the FTIR spectrum.

(1) $$3\,{\mathrm{Ca}}_{3}\,{\left({\mathrm{PO}}_{4}\right)}_{2}+H_{2}\,\text{O}\to {\mathrm{Ca}}_{9}\,\left[\left({\mathrm{HPO}}_{4}\right)\,{\left({\mathrm{PO}}_{4}\right)}_{5}\right]\,\mathrm{OH}\to$$

β-T⁢C⁢P               a⁢p⁢a⁢t⁢i⁢t⁢i⁢c⁢T⁢C⁢P

$$\text{[}\,S\,i\,n\,t\,e\,r\,i\,n\,g\,\text{]}\to 3\,{\mathrm{Ca}}_{3}\,{\left({\mathrm{PO}}_{4}\right)}_{2}$$

β-T⁢C⁢P

**FIGURE 3:**
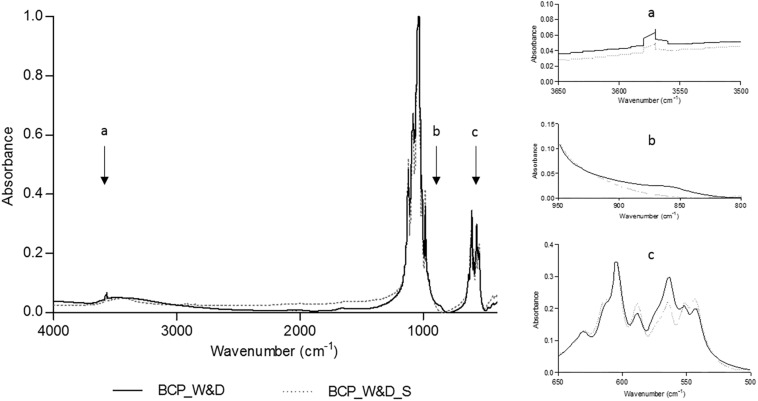
Fourier transformed infrared spectroscopy (FTIR) analyses comparing BCP_W&D and BCP_W&D_S with slight modifications at about 3575, 875, and 550 to 600 cm^–1^ reported on the graph by black arrows **(a–c)**.

The slight increase of the OH^–^ group (Figure 3a), between 3,550 and 3,600 cm^–1^ (Gibson et al., 2000; Anwer et al., 2016), could be the transformation of the β-TCP into apatitic TCP increasing the peak already existing with the 20% of HA of the original BCP sample. The clear new peak toward 875 cm^–1^ (Figure 3b) corresponds to the apparition of the HPO_4_^2–^ groups as described in the literature (Yubao et al., 1994; Anwer et al., 2016). At the same time, an evolution of the FTIR spectrum is noted between 500 and 600 cm^–1^ (Figure 3c), which correspond to HPO_4_^2–^ and PO_4_^3–^ absorption bands in apatitic chemical environments (Rey et al., 1995; Vandecandelaere et al., 2012). This sensitive method seems to clearly identify the needle-like surface layer as apatitic TCP composition. The other hypothesis, but less probable, should be the dissolution and reprecipitation of the less-soluble phase, the HA after reaction in water. In this case, as described in Eq. 2, the reaction should lead to the formation of a new calcium compound. However, no other FTIR peak apparition could prove the formation of a new compound.

(2) $${\text{Ca}}_{10}\,{\left({\mathrm{PO}}_{4}\right)}_{6}\,{\left(\mathrm{OH}\right)}_{2}+H_{2}\,\text{O}\to$$

H⁢A

$${\mathrm{Ca}}_{10-\text{x}}\,\left[{\left({\mathrm{HPO}}_{4}\right)}_{\text{x}}\,{\left({\mathrm{PO}}_{4}\right)}_{6-\text{x}}\right]\,{\text{OH}}_{2-x}+x\,{\text{Ca}}^{2+}$$

a⁢p⁢a⁢t⁢i⁢t⁢i⁢c⁢C⁢D⁢A          C⁢a⁢c⁢o⁢m⁢p⁢o⁢u⁢n⁢d

Moreover, this second hypothesis does not seem coherent with the previous results on XRD analyses (Figure 2). Indeed, the decrease of the rhombohedral phase (β-TCP) for more of a hexagonal one (apatitic form) confirms the high probability of apatitic TCP layer formation.

**FIGURE 4:**
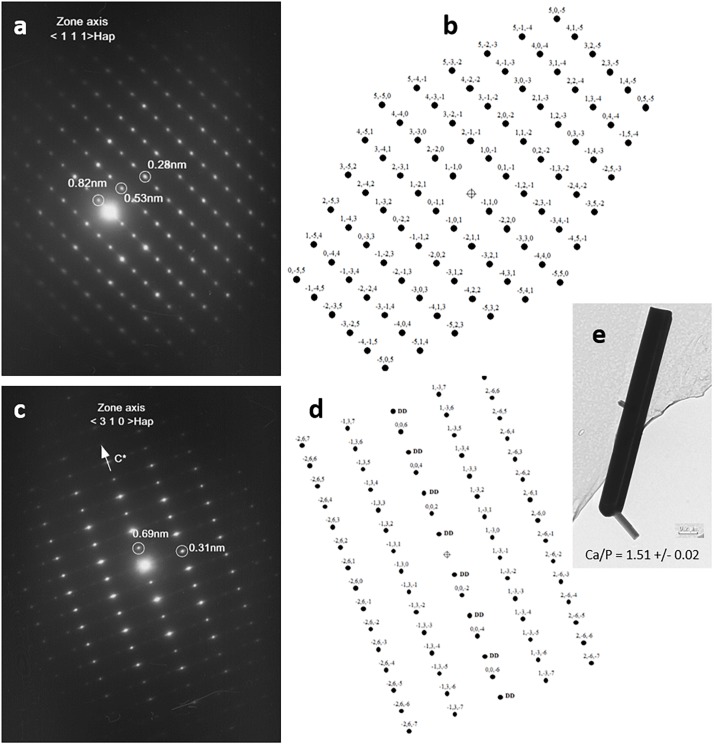
Electron diffraction patterns of needles by transmission electron microscopy (TEM) analysis **(a**,**c)** and schematic representations of reciprocal lattice of apatitic crystals according to both crystallographic orientations, <1 1 1> and < 3 1 0 > (in **b**,**d**, respectively), and bright-field picture with the atomic Ca/P ratio determined from energy-dispersive x-ray spectroscopy (EDX) quantification **(e)**.

In order to establish the crystallographic structure of needles, an electron diffraction study (SAED) was performed on several needles. Needle-like crystals were orientated with double-tilt holder in the TEM to obtain different dense planes. All needles exhibit well-crystallized ED patterns. All ED patterns were indexed according to a crystalline structure corresponding to an apatitic crystallographic cell, and none of the needles observed exhibited an ED pattern corresponding to the rhombohedral cell of β-TCP. This indicates that all observed needles have a crystallographic structure corresponding to an apatitic-like structure. Two examples are shown in Figure 4. The first one (Figure 4a) shows one ED pattern of a needle with different interplanar distances of 0.82, 0.53, and 0.28 nm and angles between these plane families of 71° and 90°, respectively with the first one, that can correspond to those of the reciprocal plane of apatitic structure with a zone axis of the electron beam orientated along the <1 1 1> crystallographic axis of the apatitic cell. The schematic representation of the 2D reciprocal lattice corresponding to the < 1 1 1 > zone axis of apatitic cell (Figure 4b) is in accordance with the ED pattern presented and gives a possible indexation of (h k l) planes at the origin of diffraction spots. The second one (Figure 4c) shows an ED pattern of needles with interplanar distances of 0.69 and 0.31 nm, with an angle of 90° between these two plane families that can correspond to those of the reciprocal plane of apatitic structure with a zone axis of the electron beam orientated along the < 3 1 0 > crystallographic axis of the apatitic cell. The schematic representation of the 2D reciprocal lattice corresponding to the < 3 1 0 > zone axis of apatitic cell (Figure 4d) is in accordance with the ED pattern presented and gives a possible indexation of (h k l) planes at the origin of diffraction spots. It should be noticed that owing to the symmetry of the crystalline cell of apatite, the diffraction spots corresponding to the (0 0 l) planes with l odd are forbidden in the reciprocal space, but spots corresponding to these indexations (0 0 l) with l odd are reignited by double diffraction phenomena for this zone axis orientation (DD in Figure 4d). The arrow in Figure 4c indicates the direction of the reciprocal crystallographic axis C^∗^ of the apatitic structure (hexagonal axis, *c* = 6.9 Å, which coincides with the axis of needles). Figure 4e shows an example of a needle. Quantitative EDX microanalyses were performed on more than 10 different needles, and the mean value of the Ca/P atomic ratio of needles determined to be 1.51 is in agreement with the chemical composition of the apatitic TCP and not with those of HA (1.67).

Rohanizadeh et al. (1998) have already shown the apatite precipitation apparition on BCP after incubation in various solutions. In a study (Tamimi et al., 2012) concerning the autoclaving of brushite into monetite-modified tomography of samples with the increase of the SSA, porosity, and interconnected macroporosity, the differences in biological properties (higher *in vitro* and *in vivo* bone regeneration properties of monetite than brushite samples) seemed to be due to the changes in the material dissolution and morphology. Other studies with TEM analyses of *in vivo* BCP implantation showed the precipitation of a clearly identified apatitic microcrystal around ceramic crystal in bone site and distributed without particular orientation in muscle sites (Rohanizadeh et al., 1999). Moreover, the authors observed that epitaxic growth of apatitic crystals seems more favorable from HA than β-TCP.

The combination of different characterization methods used in this study allowed for proving that the needle-like layer formation is composed of apatitic TCP chemical composition. This study demonstrates the capacity of MBCP^®^+ granules to generate a bioactive apatitic layer, which explains the excellent results in bone regeneration and the first choice for association in bone tissue engineering (stem cells, growth factors, and peptide). The present study shows that it is easy to control the growth of this apatitic surface layer. Further development of new bioactive medical devices are currently under progress to improve the handling properties of a bone grafts with no adverse effect on stability.

## Data Availability Statement

All datasets generated for this study are included in the article/Supplementary Material.

## Author Contributions

All authors listed have made a substantial, direct and intellectual contribution to the work, and approved it for publication.

## Conflict of Interest

CD’A and PB are employees of the Biomatlante – Advanced Medical Solutions Group plc Company.

The remaining authors declare that the research was conducted in the absence of any commercial or financial relationships that could be construed as a potential conflict of interest.

## Supplementary Material

DATA S1 Denominations of study samples.
