# Supplementary material for: Bioactivity of Biphasic Calcium Phosphate Granules, the Control of a Needle-Like Apatite Layer Formation for Further Medical Device Developments
Source: Front Bioeng Biotechnol. 2020 Jan 28;7:462. doi: 10.3389/fbioe.2019.00462 (PMC7025562; doi:10.3389/fbioe.2019.00462)
Supplement: DATA S1 — Denominations of study samples. [file Table_1.pdf]

Supplementary Material

Denominations of study samples

Low sintering temperature (first part of the study):

	Washing and Drying	Sintering
MBCP®+: Batch 0716J116	BCP1_W&D	BCP1_W&D_S
MBCP®+: Batch 0916J216	BCP2_W&D	BCP2_W&D_S
MBCP®+: Batch 1016J216	BCP3_W&D	BCP3_W&D_S

Effect of higher sintering temperature (second part of the study):

	-	Washing and Drying	Washing and storage at 37°C for 2 months
MBCP®+	BCP_0	BCP_W&D	BCP_W&37/2M
MBCP®+ 1200°C	BCP_1200	BCP_1200_W&D	BCP_1200_W&37/2M
